# Gouty Arthritis Across Ages: Understanding Disease Patterns and Predictors

**DOI:** 10.7759/cureus.58873

**Published:** 2024-04-23

**Authors:** Jaber Abdullah Alshahrani, Saleh Ali Saleh Alzahrani, Osama Saeed Ali AlGhamdi, Naif Ghormallh Ali Alzahrani, Fayez Ali Ahmed Alzahrani, Fayez S Alshehri, Abdulmajeed Saad Alshahrani, Sherefah I Alsayafi, Rayan S Alghamdi, Ghadeer Ali Alghanem, Hawra Hussain Al Radhwan, Mohannad A Alzain

**Affiliations:** 1 Family Medicine and Medical Education, Armed Forces Hospital Southern Region, Khamis Mushait, SAU; 2 Family Medicine, Alziad PHC, Ministry of Health, Al-Baha, SAU; 3 Preventive Medicine, Ministry of Health, Jeddah, SAU; 4 Family Medicine, King Fahad Armed Forces Hospital, Jeddah, SAU; 5 Family Medicine, Armed Forces Hospital Southern Region, Khamis Mushait, SAU; 6 Medicine, Najran University, Najran, SAU; 7 General Practice, Dar Al Uloom University, Riyadh, SAU; 8 Health Home Care, Aseer Central Hospital, Abha, SAU; 9 Family Medicine, Qatif Central Hospital, Qatif, SAU; 10 Family Medicine, King Fahad General Hospital, Medina, SAU; 11 Medicine, King Abdulaziz University, Jeddah, SAU

**Keywords:** al-baha region, gout disease, gout, prevalence, saudi arabia, hyperuricemia, gout crystals

## Abstract

Introduction

Gout, a chronic inflammatory joint disease, is increasingly prevalent worldwide, mainly affecting men, young females, and post-menopausal women. This study aims to investigate gout epidemiology in Al-Baha, Saudi Arabia, addressing the dearth of localized data on prevalence, risk factors, and management practices.

Methods

A cross-sectional analysis was conducted at King Fahad Hospital, Al-Baha, Saudi Arabia, covering 116 patients from March 2016 to November 2017. Data encompassed demographics, clinical presentations, and biochemical markers relevant to gout.

Results

Among 116 patients, 41 (35.3%) were diagnosed with gout, with males exhibiting a significantly higher prevalence than females (43.9% vs. 24%). Significant associations were found between gout prevalence and residency, occupational status, education level, clinical presentations (podagra, arthralgia/arthritis), and biochemical markers.

Conclusion

This study enriches global knowledge by providing localized insights into gout's epidemiology and highlighting demographic influences and clinical presentations specific to the Saudi context. The findings underscore the importance of tailored approaches in gout management, considering regional variations in prevalence, risk factors, and clinical manifestations.

## Introduction

Gout has long been recognized as among the most common chronic inflammatory joint diseases, with its prevalence steadily increasing worldwide [1,2]. Historically, it has been more prevalent in men than in women, although the incidence in women rises post-menopause and with age, approaching that in men by the age of 60 [3]. The condition is characterized by elevated serum uric acid levels (hyperuricemia), leading to the formation and deposition of urate crystals in joints, periarticular tissues, bones, and other organs [3]. These crystals trigger acute inflammatory responses, resulting in intense pain, swelling, and discomfort, particularly affecting the first metatarsophalangeal joint condition commonly referred to as "podagra" [4].

Gout can profoundly impact an individual's quality of life, impairing mobility, causing significant pain, and increasing the risk of various complications. Among these complications, kidney stones and tophi formation are notable, further adding to the burden of the disease [4,5]. Moreover, individuals with gout often experience intercortical periods characterized by asymptomatic intervals between acute attacks, during which urate crystals may continue to accumulate and cause damage. Over time, chronic gout can lead to joint deformities and irreversible damage, severely affecting the affected individual's functional abilities and overall well-being. Despite its profound impact, individuals with gout often receive inadequate education and management, exacerbating its burden on healthcare systems [6].

Despite the considerable burden imposed by gout, there are notable gaps in our understanding of the disease's epidemiology, particularly in regions outside the Western world [7]. While extensive research has been conducted in countries like the USA, UK, and other nations [8-10], comprehensive data remains on the prevalence, risk factors, and management practices related to gout in regions like Saudi Arabia, specifically in the Al-Baha region. This knowledge gap hinders the development of targeted interventions and healthcare strategies tailored to the needs of populations in these regions.

Thus, the aim of this study is to address this gap by investigating the epidemiology of gout in Al-Baha, Saudi Arabia, with a focus on understanding its prevalence, identifying independent risk factors, and describing management practices related to gout in this specific population.

## Materials and methods

Study design and setting

The study was designed as a cross-sectional analysis aimed at elucidating the prevalence, demographic patterns, and clinical manifestations of gouty arthritis among the population of Al-Baha province, Saudi Arabia. Al-Baha University Ethical Research Committee issued approval REC/COM/BU-FM/2020/0019. Data was collected from patients presenting with symptoms suggestive of gouty arthritis at King Fahad Hospital (KFH) in Al-Baha, Saudi Arabia, covering the period from March 2016 to November 2017. This temporal window provided an opportunity to assess the disease's patterns and predictors within this specific locale.

Al-Baha, situated in the southwest of Saudi Arabia, serves as the capital of the Al-Bahah Region, with a population of 533,001 individuals residing in its 31 administrative centers. KFH, located in Al Baha, offers medical services with a 14-bed capacity. This hospital setting facilitated the collection of relevant clinical data essential for the study's objectives, enabling a comprehensive analysis of gouty arthritis within the local population.

Sample size calculation

The study determined the sample size using MedCalc 15.8 software (MedCalc Software, Ostend, Belgium). It aimed to investigate the percentage of patients diagnosed with gouty arthritis. Referring to a previous study in Kuwait where the prevalence of gouty arthritis was found to be 0.8% [7], the minimum sample size required was calculated to be 73, considering a 5% alpha error, 80% study power, and 5% precision. However, the study included 116 patients based on available data.

Inclusion and exclusion criteria

The study included all patients who presented to KFH in Al-Baha for evaluation of symptoms suggestive of gouty arthritis during the study period from March 2016 to November 2017. Inclusion was not limited to those with a confirmed diagnosis of gouty arthritis via joint aspiration, identification of monosodium urate crystals, and detection through ultrasound scan for "Double Contour Sign"; it also encompassed individuals evaluated for gout but found not to have the disease, allowing for a comparative analysis between gout and non-gout cases. This approach enabled the examination of demographic and clinical characteristics across a broader spectrum of patients presenting with joint-related symptoms, facilitating a comprehensive analysis of gouty arthritis patterns and predictors. Exclusion criteria were applied to records with incomplete diagnostic information, unclear gouty arthritis status, or missing critical medical history details, ensuring the integrity of the dataset for robust comparative analysis.

Data collection procedure

Data collection was conducted through a review of electronic medical records at KFH, encompassing both patients evaluated for susceptibility to gout. This process involved the systematic extraction of detailed information on demographics, clinical presentations, diagnostic evaluations, treatment histories, and biochemical markers relevant to gout and differential diagnoses.

Variables and measures

The study incorporated an extensive array of variables, including but not limited to, patient demographics (age, sex, residency, occupational status, educational level), clinical history (prior medical history relevant to gout predisposition), clinical manifestations of gouty arthritis (affected joints, symptoms), and biochemical parameters (serum uric acid levels, renal function tests). These variables were selected for their potential to provide insights into the epidemiological characteristics of gouty arthritis and its clinical course among the studied population.

Statistical methods

Data analysis was conducted using IBM SPSS Statistics for Windows, Version 25 (Released 2017; IBM Corp., Armonk, New York, United States). Descriptive statistics (mean, standard deviation, frequencies, and percentages) were applied to characterize the study population in terms of demographic and clinical variables. Inferential statistics, including chi-square tests for categorical variables and t-tests for continuous variables, were utilized to assess differences between groups. Correlation analysis and binary logistic regression analysis were performed. Logistic regression was done to identify independent predictors of gouty arthritis prevalence, with odds ratios (ORs) and 95% confidence intervals (CIs) computed to quantify the strength of associations. A p-value of less than 0.05 was considered statistically significant for all analyses.

## Results

The study sample consisted of 116 individuals, with 41 (35.3%) diagnosed with gout. Age analysis indicated that the patients' mean age was 53.1 (SD ± 14.9). The prevalence of gout was significantly higher in males (43.9%) compared to females (24%), indicating a gender-associated risk (P = 0.03). Residency appeared to influence gout prevalence, with those living outside Al-Baha showing a higher prevalence (56.3%) than Al-Baha residents (32%). However, the difference approached but did not reach statistical significance (P = 0.08). Occupational status showed variation in gout prevalence: 44.7% for government employees, 39.3% for private sector workers, and 22% for those not working, with the observed differences nearing significance (P = 0.06). A significant association was found between educational level and gout prevalence. The highest prevalence was observed in individuals with a bachelor’s degree or higher (50%), followed by those with elementary degrees (33.3%), illiterates (31.3%), and secondary degree holders (7.1%) (P = 0.01), as shown in Table 1.

**Table 1:** Demographic characteristics of study participants *: Statistically significant

Variables		Total	Gout N (%)	p-value
Age in years (mean (SD))		53.1 (14.9)		
Sex	Male	66	29 (43.9)	0.03*
	Female	50	12 (24)	
Residency	Al-Baha	100	32 (32)	0.08
	Outside Al-Baha	16	9 (56.3)	
Occupation	Working in government sector	47	21 (44.7)	0.06
	Working in private sector	28	11 (39.3)	
	Not working	41	9 (22)	
Educational level	Illiterate	16	5 (31.3)	0.01*
	Elementary degree	12	4 (33.3)	
	Secondary degree	28	2 (7.1)	
	Bachelor degree and higher	60	30 (50)	
Past history of metabolic syndrome	Positive	5	3 (60)	0.32
	Negative	111	38 (34.2)	
History of diuretic use	Positive	19	9 (47.4)	0.21
	Negative	97	32 (33)	
Past history of renal disease	Positive	15	9 (60)	0.04*
	Negative	101	32 (31.7)	
Family history of gouty arthritis	Positive	59	30 (50.8)	<0.01*
	Negative	57	11 (19.3)	
Past history of diabetes mellitus	Positive	32	18 (56.3)	<0.01*
	Negative	84	23 (27.4)	
Past history of hypertension	Positive	30	14 (46.7)	0.11
	Negative	86	27 (31.4)	

In terms of clinical presentations, significant differences were noted. Podagra was reported by 85.7% of gout sufferers, arthralgia and arthritis combination by 58.6%, isolated arthralgia by 25%, edema of the joints by 16.7%, and no cases of arthritis alone in the gout group (P < 0.01). The ankle was the most affected joint in 60% of cases, followed by the big toe (34.9%), and the knee (18.6%), with significant differences observed (P < 0.01). Biochemical analyses revealed significantly higher levels of serum uric acid, blood urea nitrogen, and creatinine in the gout group compared to non-gout individuals (P < 0.01 for all) as shown in Table 2.

**Table 2:** Comparison of biochemical parameters and clinical picture among study participants *: Statistically significant

Variables		Non-gout N (%)	Gout N (%)	p-value
Clinical presentation	Arthralgia	51 (75)	17 (25)	<0.01*
	Edema of the joints	5 (83.3)	1 (16.7)	
	Arthritis	6 (100)	0	
	Podagra	1 (14.3)	6 (85.7)	
	Arthralgia and arthritis	12 (41.4)	17 (58.6)	
Most affected joint	Big toe	28 (65.1)	15 (34.9)	<0.01*
	Ankle	12 (40)	18 (60)	
	Knee	35 (81.4)	8 (18.6)	
Serum uric acid ​​​​​​"*mg/meq"* (mean ± SD)		456 (41)	601 (74)	<0.01*
Blood urea nitrogen ​​​​​​"*mg/meq"* (mean ± SD)		7.1 (1.5)	8 (1.9)	0.01*
Creatinine ​​​​​​"*mg/meq"* (mean ± SD)		99 (30)	131 (45)	<0.01*

**Figure 1:**
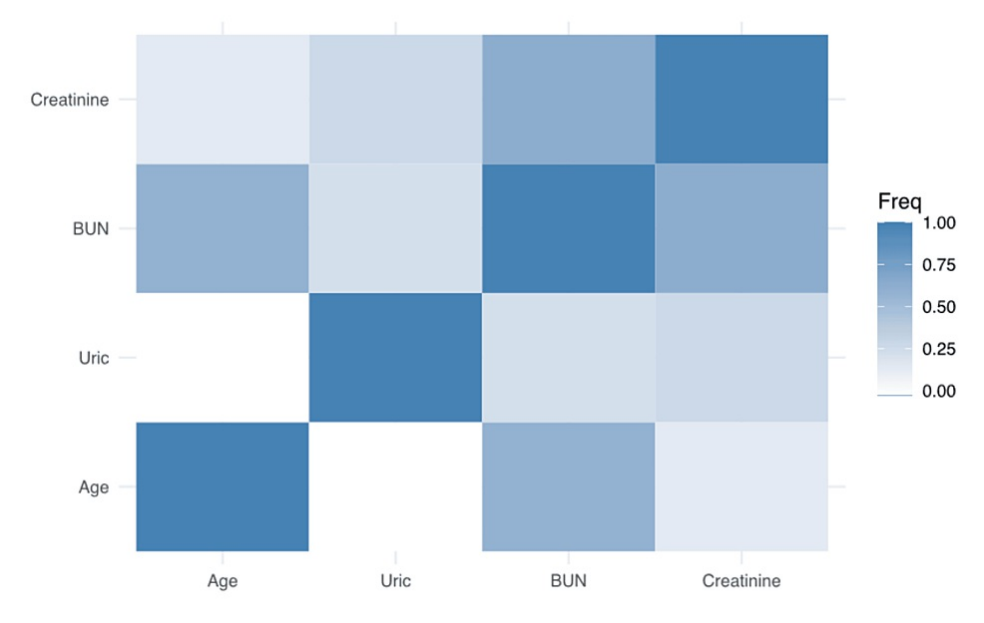
Correlation heatmap of age, uric acid, BUN, and creatinine levels among study participants BUN: Blood urea nitrogen

Significant correlations were observed between several pairs of variables. Specifically, a statistically significant positive correlation was found between blood urea nitrogen (BUN) and creatinine (r = 0.714, p < 0.001), as well as between uric acid and creatinine (r = 0.235, p = 0.011). Additionally, there was a significant negative correlation between age and BUN (r = -0.097, p = 0.3) (Figure 1).

Binary logistic regression analysis identified several factors significantly associated with an increased prevalence of gout. Being male was associated with a higher prevalence of gout compared to females (OR = 0.4, P = 0.02). A positive past history of renal disease (OR = 3.2, P = 0.03), a family history of gouty arthritis (OR = 0.2, P = 0.01), and a past history of diabetes mellitus (OR = 3.4, P = 0.005) were significantly associated with gout prevalence. Clinical presentations of podagra (OR = 18, P = 0.01) and the combination of arthralgia and arthritis (OR = 4.2, P = 0.002) were strongly linked to gout. Furthermore, having the big toe as the most affected joint significantly increased the risk of gout (OR = 6.5, P = 0.01) (Table 3).

**Table 3:** Binary logistic regression analysis of factors associated with gout prevalence *: Statistically significant

Variable		Univariate regression	
		OR 95% (CI)	p-value
Sex	Male	1 (r)	0.02*
	Female	0.4 (0.1-0.9)	
Educational level	Illiterate	1 (r)	
	Elementary degree	1.1 (0.2-5.4)	0.93
	Secondary degree	0.1 (0.02-0.99)	0.05*
	Bachelor degree and higher	2.2 (0.6-7.1)	0.12
Past history of renal disease	Negative	1 (r)	0.03*
	Positive	3.2 (1.1-9.8)	
Family history of gouty arthritis	Negative	1 (r)	0.01
	Positive	0.2 (0.1-0.5)	
Past history of diabetes mellitus	Negative	1 (r)	0.005*
	Positive	3.4 (1.4-7.9)	
Clinical presentation	Arthralgia	1 (r)	
	Edema of the joints	0.6 (0.06-5.5)	0.61
	Podagra	18 (2-160)	0.01*
	Arthralgia and arthritis	4.2 (1.6-10.6)	0.002*
Most affected joint	Knee	1 (r)	
	Ankle	2.3 (0.8-6.3)	0.09
	Big toe	6.5 (2.2-18.9)	0.01*

## Discussion

This cross-sectional study, with its unique focus on gouty arthritis among a sample of individuals in Saudi Arabia, has revealed several novel findings. These include insights into the prevalence, demographic influences, and clinical presentations associated with the disease. By enriching the existing body of research, this study provides localized insights into the epidemiology of gout, offering a fresh perspective for comparison with global and regional studies on the condition.

Our findings present a nuanced picture of gout prevalence across different age groups, challenging the commonly held perception that gout primarily affects middle-aged and older individuals. However, the absence of statistical significance in age-related prevalence differences (P = 0.2) highlights the need for further investigation with larger sample sizes to clarify age as a determinant of gout risk.

Gender emerged as a significant predictor, with males exhibiting a higher prevalence of gout than females (43.9% vs. 24%, P = 0.03). This observation, consistent with previous studies, underscores the importance of gender-specific approaches in the prevention, diagnosis, and management of gout. Such disparities in gout prevalence have been widely documented, including in the extensive epidemiological review by Han et al. [10].

A significant association between educational level and gout prevalence was identified, with the highest prevalence observed in individuals holding a bachelor's degree or higher. This intriguing finding contrasts with previous studies that associated lower socioeconomic status and education levels with increased gout risk [11,12]. It may reflect lifestyle differences, dietary habits, or healthcare access among different educational groups in Saudi Arabia.

Clinical presentations of gout in our study population were predominantly characterized by podagra, followed by a combination of arthralgia and arthritis. The significant association of these clinical features with gout, alongside the absence of arthritis alone, provides valuable insights for clinicians in diagnosing and managing gout [13,14]. The logistic regression analysis further identified male gender, a positive past history of renal disease, a family history of gouty arthritis, and a past history of diabetes mellitus as significant predictors of gout prevalence. Notably, the presence of podagra and having the big toe as the most affected joint emerged as strong predictors, emphasizing the importance of these clinical features in assessing gout risk.

The strong associations between elevated serum uric acid levels, blood urea nitrogen, and creatinine with gout in our cohort echo findings from other regions, highlighting the pivotal role of kidney function and metabolic health in gout pathophysiology. This is supported by the association studies by Alshahrani et al. [13] and Weaver et al. [14], which underscore the interconnectedness of metabolic dysfunctions with hyperuricemia and gout [9,15]. These findings highlight the importance of comprehensive biochemical assessment in individuals at risk of or suffering from gout.

Our study's context within Saudi Arabia offers a valuable comparison point to the global and regional epidemiology of gout, contributing to the understanding of gout's distribution in the Middle East region, which is not as thoroughly represented in global epidemiology reviews [10,16]. The observed variations in prevalence rates across genders, age groups, and socio-economic backgrounds within our study population underscore the multifaceted nature of gout. This complexity is reflected in the diverse prevalence rates and risk factors identified across different populations worldwide. For instance, the high prevalence rates in Pacific Islanders and Maori, with studies in Australasia reporting gout prevalence rates of 8.8% for Maori males and 7.0% in Australian Aboriginal men, starkly contrast with the lower rates observed in European cohorts, where countries like the UK and Germany reported gout prevalences of 1.4%, and the highlands of Scotland saw a mere 0.34% prevalence [17,18]. Notably, in black Africans, where gout was once considered rare, varied prevalence rates from 0.03% in Nigerian men to 1.9% in Togolese patients highlight the geographical and ethnic diversity in gout distribution [19,20]. Similarly, in Asia, the prevalence ranged significantly, from 0.16% in rural Taiwanese populations to an astonishing 11.7% in Taiwanese aborigines, indicating stark differences even within the same country [21,22].

These global comparisons illuminate the complexity of gout's epidemiology, showing that prevalence can be influenced by a variety of factors, including genetic predispositions, lifestyle, and socio-economic status. Contrasting these rates with those from other regions emphasizes the importance of localized research in understanding gout's multifactorial nature and the need for tailored approaches to its management and prevention [10].

The synthesis of our findings with the existing literature emphasizes the necessity of a multidimensional approach to addressing gout. The demographic patterns, clinical presentations, and associated risk factors identified in our study and others underline the importance of considering genetic predispositions, lifestyle factors, and socio-economic conditions in both the prevention and management of gout. Moreover, the regional insights provided by our study, set against the backdrop of global epidemiology, underscore the significance of localized research in understanding and combating gout. By aligning our observations with those from other regions and populations, we can better appreciate the global burden of gout while recognizing the specific challenges and opportunities present in Saudi Arabia.

The study's limitations include its nature as a cross-sectional design, which precludes causal inference, and the relatively small sample size and single-center setting, which may limit the generalizability of findings to broader populations within Saudi Arabia.

## Conclusions

Our study provides a comprehensive contribution to the epidemiological understanding of gout in Saudi Arabia. It reveals intricate associations between demographic factors, socioeconomic status, clinical presentations, and biochemical markers, corroborating the gender disparity in gout prevalence seen globally. The study uncovers the nuanced influences of residency, occupational status, and education level on disease prevalence. Through a detailed examination of clinical manifestations and identification of vital biochemical markers associated with gout, this research aligns with global findings. It highlights unique aspects of the disease within the Saudi context. By integrating local epidemiological data with broader global insights, this study lays a foundation for future targeted interventions and research focused on mitigating gout's impact in Saudi Arabia and beyond.
